# Supplementary material for: A Likelihood Approach for Real-Time Calibration of Stochastic Compartmental Epidemic Models
Source: PLoS Comput Biol. 2017 Jan 17;13(1):e1005257. doi: 10.1371/journal.pcbi.1005257 (PMC5240920; doi:10.1371/journal.pcbi.1005257)
Supplement: S1 File — (TAR.GZ) [file pcbi.1005257.s014.tar.gz › HSPH_Online-SI-Revision/Additions/IPoi10/n100-extreme-SEITR/n100-extreme-SEITR_BENCH_table8.pdf]

| | | | | | | | | | | | | | | | | | | | | | | | | | | | |
|---|---|---|---|---|---|---|---|---|---|---|---|---|---|---|---|---|---|---|---|---|---|---|---|---|---|---|---|
| 1 | R0 | {1.38654, 9.85565} | {2.97157, 19.3547} | {{2.97157, 19.3547}, {2.97157, 19.3547}} | {114.315, 96.3818} | inf | 78. | 4571.3 | {2273., 7678.} | 594.726 | 0.789964 | newly | 382. | 1440.89 | {692., 2346.} | 277.196 | 0.524889 | Reff | 0.108483 | 0.217639 | {0.197996, 0.240994} | 100.62 | ar | 92 379. | 93 857. | {93 718., 94 006.} | 1.59988 |
| 2 | R0 | {2.36629, 12.2029} | {2.9884, 12.8326} | {{2.9884, 12.8326}, {2.9884, 12.8326}} | {26.2905, 5.16025} | inf | 5. | 2268.38 | {1158., 4048.} | 229.229 | 0.45373 | newly | 300. | 1004.42 | {503., 1779.} | 234.805 | 0.459721 | Reff | 0.0235682 | 0.196303 | {0.186506, 0.205841} | 732.915 | ar | 99 022. | 93 972. | {93 820., 94 122.} | 5.09989 |
| 3 | R0 | {2.32253, 12.836} | {2.9194, 10.1535} | {{2.9194, 10.1535}, {2.9194, 10.1535}} | {25.6991, 20.8983} | inf | 20. | 651.967 | {235., 1075.} | 53.2988 | 0.346708 | newly | 463. | 344.25 | {136., 583.} | 50.7595 | 0.291672 | Reff | 0.0245491 | 0.19456 | {0.189294, 0.200534} | 692.531 | ar | 98 997. | 93 492.2 | {93 345., 93 642.} | 5.56061 |
| 4 | R0 | {1.79075, 11.2058} | {2.9884, 12.8326} | {{2.9884, 12.8326}, {2.9884, 12.8326}} | {66.8798, 14.5175} | inf | 31. | 1490.06 | {754., 2399.} | 84.0097 | 0.219254 | newly | 412. | 653.6 | {337., 1076.} | 66.0493 | 0.182102 | Reff | 0.0574114 | 0.190422 | {0.183189, 0.19571} | 231.679 | ar | 96 920. | 93 970. | {93 809., 94 134.} | 3.0437 |
| 5 | R0 | {2.26952, 9.62371} | {2.98388, 9.60029} | {{2.98388, 9.60029}, {2.98388, 9.60029}} | {31.4763, 0.243357} | inf | 0. | 521.369 | {221., 849.} | 178.824 | 0.354283 | newly | 78. | 285.631 | {112., 463.} | 266.193 | 0.471717 | Reff | 0.0257137 | 0.184396 | {0.179719, 0.188372} | 617.111 | ar | 98 881. | 93 938.1 | {93 835., 94 062.} | 4.9988 |
| 6 | R0 | {1.57191, 6.89004} | {2.9194, 10.1535} | {{2.9194, 10.1535}, {2.9194, 10.1535}} | {85.7231, 47.3649} | inf | 5. | 537.015 | {263., 947.} | 894.473 | 0.92721 | newly | 40. | 284.154 | {142., 510.} | 610.385 | 0.780138 | Reff | 0.077951 | 0.194482 | {0.18944, 0.198636} | 149.492 | ar | 95 046. | 93 464.4 | {93 338., 93 607.} | 1.66408 |
| 7 | R0 | {1.99226, 17.2339} | {2.97157, 19.3547} | {{2.97157, 19.3547}, {2.97157, 19.3547}} | {49.1557, 12.306} | inf | 78. | 4598.11 | {2158., 9066.} | 75.1369 | 0.198836 | newly | 982. | 1474.19 | {659., 2843.} | 70.077 | 0.194314 | Reff | 0.0456427 | 0.255231 | {0.196064, 0.259091} | 459.195 | ar | 97 992. | 93 863.7 | {93 739., 93 993.} | 4.21289 |
| 8 | R0 | {2.63369, 15.1879} | {2.9884, 12.8326} | {{2.9884, 12.8326}, {2.9884, 12.8326}} | {13.4682, 15.5077} | inf | 5. | 2545.07 | {1269., 4496.} | 82.2901 | 0.198013 | newly | 510. | 1124. | {594., 1896.} | 120.952 | 0.273385 | Reff | 0.0154861 | 0.198926 | {0.187163, 0.21379} | 1184.55 | ar | 99 451. | 93 961.3 | {93 830., 94 115.} | 5.52006 |
| 9 | R0 | {2.53271, 9.90552} | {2.98388, 9.60029} | {{2.98388, 9.60029}, {2.98388, 9.60029}} | {17.8137, 3.08141} | inf | 2. | 486.597 | {256., 837.} | 185.388 | 0.413119 | newly | 96. | 269.472 | {140., 469.} | 181.51 | 0.405406 | Reff | 0.0167159 | 0.184948 | {0.179451, 0.189864} | 1006.42 | ar | 99 355. | 93 909.4 | {93 782., 94 067.} | 5.48091 |
| 10 | R0 | {2.48791, 18.9003} | {2.95376, 15.278} | {{2.95376, 15.278}, {2.95376, 15.278}} | {18.7246, 19.1653} | inf | 42. | 2284.13 | {1137., 3906.} | 44.4311 | 0.281978 | newly | 1167. | 879.411 | {441., 1507.} | 38.6293 | 0.218422 | Reff | 0.0212716 | 0.201768 | {0.190902, 0.215418} | 848.532 | ar | 99 291. | 93 721.2 | {93 542., 93 862.} | 5.60961 |
| 11 | R0 | {2.84414, 18.4765} | {2.86118, 15.5722} | {{2.86118, 15.5722}, {2.86118, 15.5722}} | {0.599127, 15.7189} | inf | 13. | 2680.17 | {1592., 3990.} | 27.8434 | 0.141354 | newly | 1003. | 1016.67 | {612., 1502.} | 28.5809 | 0.123925 | Reff | 0.0117463 | 0.220847 | {0.21127, 0.230897} | 1780.14 | ar | 99 654. | 93 001.6 | {92 801., 93 143.} | 6.67548 |
| 12 | R0 | {2.29369, 14.622} | {2.7943, 13.5962} | {{2.7943, 13.5962}, {2.7943, 13.5962}} | {21.8255, 7.01546} | inf | 14. | 3498.8 | {1401., 6479.} | 158.555 | 0.336309 | newly | 542. | 1496.1 | {588., 2738.} | 177.4 | 0.362791 | Reff | 0.0247948 | 0.24093 | {0.219157, 0.269371} | 871.696 | ar | 98 982. | 92 448.1 | {92 245., 92 634.} | 6.60106 |
| 13 | R0 | {2.73413, 16.7529} | {2.99939, 13.7076} | {{2.99939, 13.7076}, {2.99939, 13.7076}} | {9.70181, 18.1777} | inf | 10. | 1738.84 | {1021., 2592.} | 35.814 | 0.173716 | newly | 706. | 725.109 | {420., 1119.} | 34.271 | 0.138666 | Reff | 0.0138347 | 0.19057 | {0.183263, 0.1973} | 1277.48 | ar | 99 543. | 94 046.7 | {93 932., 94 181.} | 5.5215 |
| 14 | R0 | {1.79554, 16.5943} | {2.76795, 19.6041} | {{2.76795, 19.6041}, {2.76795, 19.6041}} | {54.157, 18.1376} | inf | 185. | 5312.28 | {2325., 8399.} | 60.6059 | 0.194133 | newly | 1313. | 1677.41 | {723., 2631.} | 53.6037 | 0.18635 | Reff | 0.0662734 | 0.267007 | {0.236355, 0.296198} | 302.887 | ar | 96 821. | 92 174. | {92 010., 92 374.} | 4.79955 |
| 15 | R0 | {1.91429, 13.7771} | {2.97157, 19.3547} | {{2.97157, 19.3547}, {2.97157, 19.3547}} | {55.2309, 40.4846} | inf | 37. | 3944.25 | {1910., 6024.} | 168.499 | 0.368634 | newly | 621. | 1241.62 | {588., 1830.} | 102.869 | 0.253669 | Reff | 0.0501544 | 0.21396 | {0.196956, 0.228217} | 326.604 | ar | 97 541. | 93 838.9 | {93 695., 93 973.} | 3.79541 |
| 16 | R0 | {1.42094, 10.5871} | {2.8939, 16.8013} | {{2.8939, 16.8013}, {2.8939, 16.8013}} | {103.661, 58.696} | inf | 126. | 3168.45 | {1537., 6163.} | 163.942 | 0.346419 | newly | 644. | 1124.83 | {524., 2224.} | 82.0135 | 0.201439 | Reff | 0.103388 | 0.21974 | {0.204917, 0.240657} | 112.54 | ar | 93 044. | 93 283.6 | {93 140., 93 473.} | 0.259913 |
| 17 | R0 | {1.45515, 6.16466} | {2.9194, 10.1535} | {{2.9194, 10.1535}, {2.9194, 10.1535}} | {100.625, 64.7049} | inf | 5. | 1094.46 | {506., 2055.} | 2088.92 | 1.26078 | newly | 41. | 575.369 | {258., 1065.} | 1303.34 | 1.06737 | Reff | 0.0982954 | 0.198635 | {0.191892, 0.20573} | 102.079 | ar | 93 254. | 93 468.9 | {93 258., 93 615.} | 0.235948 |
| 18 | R0 | {2.85669, 13.2416} | {2.83858, 10.6973} | {{2.83858, 10.6973}, {2.83858, 10.6973}} | {0.63395, 19.2144} | inf | 1. | 1499.32 | {590., 2890.} | 102.385 | 0.246469 | newly | 314. | 771.2 | {304., 1512.} | 148.8 | 0.316884 | Reff | 0.0106269 | 0.214305 | {0.20662, 0.223794} | 1916.63 | ar | 99 644. | 92 853.2 | {92 736., 93 001.} | 6.81506 |
| 19 | R0 | {2.56417, 17.9558} | {2.8939, 16.8013} | {{2.8939, 16.8013}, {2.8939, 16.8013}} | {12.8591, 6.42968} | inf | 35. | 5991.62 | {2913., 10 695.} | 91.877 | 0.237314 | newly | 1136. | 2138.69 | {1010., 3822.} | 92.9491 | 0.237989 | Reff | 0.01818 | 0.246603 | {0.214178, 0.293847} | 1256.45 | ar | 99 389. | 93 289.5 | {93 152., 93 480.} | 6.13704 |
| 20 | R0 | {2.27517, 11.1864} | {2.92375, 10.9639} | {{2.92375, 10.9639}, {2.92375, 10.9639}} | {28.5069, 1.98902} | inf | 9. | 1084.65 | {552., 1996.} | 136.304 | 0.314834 | newly | 219. | 537.661 | {277., 998.} | 145.905 | 0.332666 | Reff | 0.0255502 | 0.198228 | {0.192792, 0.203873} | 675.84 | ar | 98 894. | 93 482.9 | {93 348., 93 632.} | 5.4716 |
| 21 | R0 | {2.35735, 10.4735} | {2.9194, 10.1535} | {{2.9194, 10.1535}, {2.9194, 10.1535}} | {23.8425, 3.05533} | inf | 3. | 980.734 | {505., 1725.} | 228.005 | 0.464806 | newly | 156. | 520.719 | {269., 929.} | 233.794 | 0.471879 | Reff | 0.0230549 | 0.197458 | {0.191192, 0.204358} | 756.47 | ar | 99 036. | 93 470.5 | {93 330., 93 630.} | 5.61971 |
| 22 | R0 | {1.79551, 11.9179} | {2.7943, 13.5962} | {{2.7943, 13.5962}, {2.7943, 13.5962}} | {55.6271, 14.0822} | inf | 31. | 1376.01 | {587., 2623.} | 74.0359 | 0.211987 | newly | 400. | 581.917 | {243., 1087.} | 67.7153 | 0.204892 | Reff | 0.0555172 | 0.221116 | {0.212786, 0.231312} | 298.283 | ar | 97 033. | 92 470.3 | {92 329., 92 625.} | 4.70224 |
| 23 | R0 | {1.23545, 6.31687} | {2.9884, 12.8326} | {{2.9884, 12.8326}, {2.9884, 12.8326}} | {141.888, 103.148} | inf | 19. | 1617.7 | {876., 2884.} | 1534.04 | 1.15846 | newly | 79. | 717.048 | {386., 1267.} | 807.655 | 0.902551 | Reff | 0.139655 | 0.191387 | {0.183518, 0.199924} | 37.0422 | ar | 88 739. | 93 965.9 | {93 818., 94 116.} | 5.8902 |
| 24 | R0 | {2.24089, 10.1789} | {2.83858, 10.6973} | {{2.83858, 10.6973}, {2.83858, 10.6973}} | {26.672, 5.09289} | inf | 7. | 1342.38 | {556., 2831.} | 286.854 | 0.505944 | newly | 171. | 686.083 | {283., 1485.} | 301.218 | 0.519378 | Reff | 0.025703 | 0.214236 | {0.204974, 0.225695} | 733.504 | ar | 98 868. | 92 819.1 | {92 624., 92 996.} | 6.11821 |
| 25 | R0 | {1.57298, 10.2607} | {2.86118, 15.5722} | {{2.86118, 15.5722}, {2.86118, 15.5722}} | {81.8955, 51.7655} | inf | 47. | 4184. | {2632., 5954.} | 380.92 | 0.65549 | newly | 443. | 1589.26 | {946., 2259.} | 258.75 | 0.527083 | Reff | 0.0819837 | 0.233368 | {0.216763, 0.249323} | 184.652 | ar | 94 957. | 93 018.3 | {92 856., 93 161.} | 2.0417 |
| 26 | R0 | {1.2154, 8.2576} | {2.8939, 16.8013} | {{2.8939, 16.8013}, {2.8939, 16.8013}} | {138.103, 103.465} | inf | 88. | 4645.9 | {2092., 9331.} | 784.934 | 0.862628 | newly | 345. | 1667.62 | {740., 3383.} | 383.368 | 0.596928 | Reff | 0.143952 | 0.235304 | {0.2081, 0.274863} | 63.4603 | ar | 88 421. | 93 262.3 | {93 103., 93 413.} | 5.47534 |
| 27 | R0 | {2.22841, 18.7983} | {2.8833, 17.1847} | {{2.8833, 17.1847}, {2.8833, 17.1847}} | {29.3882, 8.58375} | inf | 67. | 2650.05 | {1254., 4156.} | 46.3185 | 0.246594 | newly | 1079. | 928.939 | {461., 1419.} | 44.8676 | 0.215079 | Reff | 0.0322005 | 0.217624 | {0.203936, 0.229828} | 575.84 | ar | 98 756. | 93 182.2 | {93 038., 93 335.} | 5.64403 |
| 28 | R0 | {1.27778, 5.36767} | {2.98388, 9.60029} | {{2.98388, 9.60029}, {2.98388, 9.60029}} | {133.521, 78.854} | inf | 7. | 288.348 | {136., 394.} | 748.084 | 0.840308 | newly | 32. | 157.924 | {77., 228.} | 393.513 | 0.605118 | Reff | 0.130065 | 0.182637 | {0.178048, 0.186284} | 40.4199 | ar | 89 843. | 93 944.1 | {93 822., 94 079.} | 4.56473 |
| 29 | R0 | {2.38042, 15.8901} | {2.99939, 13.7076} | {{2.99939, 13.7076}, {2.99939, 13.7076}} | {26.0026, 13.735} | inf | 37. | 1236.37 | {557., 1906.} | 60.7685 | 0.399871 | newly | 849. | 517.317 | {230., 802.} | 57.2536 | 0.350921 | Reff | 0.0237804 | 0.187731 | {0.179993, 0.192681} | 689.437 | ar | 99 112. | 94 019.2 | {93 890., 94 159.} | 5.13845 |
| 30 | R0 | {2.53188, 12.7607} | {2.9194, 10.1535} | {{2.9194, 10.1535}, {2.9194, 10.1535}} | {15.3056, 20.4315} | inf | 11. | 1102.77 | {490., 1957.} | 52.8617 | 0.195128 | newly | 428. | 586.609 | {251., 1055.} | 61.5739 | 0.178086 | Reff | 0.0182295 | 0.198281 | {0.191046, 0.205321} | 987.689 | ar | 99 317. | 93 480.7 | {93 316., 93 645.} | 5.87647 |
| 31 | R0 | {1.24482, 10.5471} | {2.86095, 19.9929} | {{2.86095, 19.9929}, {2.86095, 19.9929}} | {129.828, 89.5583} | inf | 170. | 4843.46 | {2402., 7246.} | 278.395 | 0.524518 | newly | 689. | 1497.41 | {728., 2249.} | 118.364 | 0.28572 | Reff | 0.142009 | 0.240433 | {0.217003, 0.262092} | 69.3084 | ar | 89 097. | 93 029.3 | {92 882., 93 189.} | 4.41346 |
| 32 | R0 | {2.30446, 17.9607} | {2.7943, 13.5962} | {{2.7943, 13.5962}, {2.7943, 13.5962}} | {21.2562, 24.3003} | inf | 48. | 2070.09 | {938., 3686.} | 50.0666 | 0.309535 | newly | 1094. | 877.966 | {405., 1535.} | 44.6196 | 0.232138 | Reff | 0.0266626 | 0.227134 | {0.216614, 0.239248} | 751.883 | ar | 99 028. | 92 461.3 | {92 321., 92 596.} | 6.63111 |
| 33 | R0 | {2.31579, 11.8236} | {2.98388, 9.60029} | {{2.98388, 9.60029}, {2.98388, 9.60029}} | {28.8493, 18.804} | inf | 5. | 147. | {64., 279.} | 71.387 | 0.612938 | newly | 197. | 80.0323 | {34., 141.} | 66.4156 | 0.508133 | Reff | 0.0234821 | 0.182719 | {0.176138, 0.188283} | 678.121 | ar | 99 012. | 93 910.1 | {93 715., 94 126.} | 5.15285 |
| 34 | R0 | {1.6678, 10.6744} | {2.96307, 14.3219} | {{2.96307, 14.3219}, {2.96307, 14.3219}} | {77.6634, 34.1705} | inf | 36. | 1895.46 | {961., 2914.} | 231.374 | 0.46443 | newly | 278. | 769.492 | {384., 1227.} | 176.796 | 0.384919 | Reff | 0.0712985 | 0.198228 | {0.18774, 0.208422} | 178.026 | ar | 95 809. | 93 768.7 | {93 656., 93 912.} | 2.12956 |
| 35 | R0 | {1.65476, 7.07559} | {2.98388, 9.60029} | {{2.98388, 9.60029}, {2.98388, 9.60029}} | {80.321, 35.6818} | inf | 5. | 515.679 | {255., 767.} | 647.36 | 0.786645 | newly | 50. | 281.411 | {135., 412.} | 462.821 | 0.662223 | Reff | 0.0705259 | 0.183883 | {0.178764, 0.188969} | 160.731 | ar | 95 746. | 93 955.7 | {93 803., 94 111.} | 1.86988 |
| 36 | R0 | {2.71807, 18.5959} | {2.95376, 15.278} | {{2.95376, 15.278}, {2.95376, 15.278}} | {8.67123, 17.8421} | inf | 24. | 5091.64 | {2426., 9439.} | 54.2369 | 0.173471 | newly | 1211. | 1968.73 | {925., 3645.} | 71.0924 | 0.196022 | Reff | 0.0154386 | 0.225836 | {0.202185, 0.263239} | 1362.8 | ar | 99 540. | 93 731.7 | {93 564., 93 859.} | 5.8351 |
| 37 | R0 | {2.6202, 12.7552} | {2.99939, 13.7076} | {{2.99939, 13.7076}, {2.99939, 13.7076}} | {14.4718, 7.46676} | inf | 9. | 2417.21 | {1228., 4065.} | 176.885 | 0.399038 | newly | 383. | 1010.31 | {511., 1685.} | 163.788 | 0.377453 | Reff | 0.016088 | 0.195195 | {0.185572, 0.206748} | 1113.3 | ar | 99 426. | 94 063.9 | {93 904., 94 209.} | 5.39301 |
| 38 | R0 | {1.47672, 9.81651} | {2.86118, 15.5722} | {{2.86118, 15.5722}, {2.86118, 15.5722}} | {93.7524, 58.6328} | inf | 40. | 3393.58 | {1254., 6754.} | 496.411 | 0.667837 | newly | 323. | 1309.35 | {485., 2565.} | 305.371 | 0.496536 | Reff | 0.0934468 | 0.230007 | {0.206005, 0.257077} | 146.137 | ar | 93 815. | 93 011.5 | {92 820., 93 190.} | 0.85645 |
| 39 | R0 | {1.90004, 12.3313} | {2.7943, 13.5962} | {{2.7943, 13.5962}, {2.7943, 13.5962}} | {47.0653, 10.2576} | inf | 30. | 2486.9 | {1057., 4646.} | 136.739 | 0.30979 | newly | 464. | 1054.39 | {464., 1990.} | 129.679 | 0.297641 | Reff | 0.0517191 | 0.231372 | {0.217536, 0.248665} | 347.363 | ar | 97 413. | 92 447. | {92 283., 92 620.} | 5.09792 |
| 40 | R0 | {2.64218, 18.5237} | {2.99939, 13.7076} | {{2.99939, 13.7076}, {2.99939, 13.7076}} | {13.5195, 25.9997} | inf | 25. | 1284.96 | {726., 2195.} | 56.3726 | 0.390027 | newly | 874. | 532.119 | {296., 893.} | 46.6 | 0.286588 | Reff | 0.0162494 | 0.187438 | {0.180263, 0.193431} | 1053.5 | ar | 99 474. | 94 042.6 | {93 876., 94 186.} | 5.46014 |
| 41 | R0 | {1.6945, 7.17996} | {2.9194, 10.1535} | {{2.9194, 10.1535}, {2.9194, 10.1535}} | {72.2868, 41.4144} | inf | 4. | 1089.25 | {461., 1807.} | 957.521 | 0.913446 | newly | 74. | 580.058 | {242., 1005.} | 683.862 | 0.780578 | Reff | 0.0626626 | 0.198357 | {0.190491, 0.206781} | 216.547 | ar | 96 332. | 93 480.9 | {93 349., 93 639.} | 2.95965 |
| 42 | R0 | {1.84559, 18.0074} | {2.93978, 19.9817} | {{2.93978, 19.9817}, {2.93978, 19.9817}} | {59.2867, 10.9638} | inf | 180. | 5389.89 | {2861., 9035.} | 50.4079 | 0.15436 | newly | 1374. | 1663.03 | {849., 2808.} | 44.9547 | 0.153592 | Reff | 0.0586713 | 0.233831 | {0.207049, 0.262875} | 298.543 | ar | 97 358. | 93 601.5 | {93 453., 93 748.} | 3.85848 |
| 43 | R0 | {2.24965, 17.0361} | {2.95376, 15.278} | {{2.95376, 15.278}, {2.95376, 15.278}} | {31.2986, 10.3199} | inf | 59. | 2629.4 | {955., 4707.} | 60.4849 | 0.301346 | newly | 1018. | 1014.81 | {346., 1797.} | 62.8221 | 0.265135 | Reff | 0.0319 | 0.206032 | {0.190252, 0.22549} | 545.867 | ar | 98 770. | 93 730.9 | {93 571., 93 887.} | 5.10185 |
| 44 | R0 | {2.54324, 15.7967} | {2.7943, 13.5962} | {{2.7943, 13.5962}, {2.7943, 13.5962}} | {9.87166, 13.9301} | inf | 13. | 3123.22 | {1481., 5588.} | 97.089 | 0.233635 | newly | 626. | 1332.71 | {617., 2383.} | 115.077 | 0.264256 | Reff | 0.0177772 | 0.236163 | {0.21966, 0.255259} | 1228.46 | ar | 99 354. | 92 484.5 | {92 344., 92 665.} | 6.91413 |
| 45 | R0 | {2.54943, 14.8032} | {2.9884, 12.8326} | {{2.9884, 12.8326}, {2.9884, 12.8326}} | {17.2184, 13.312} | inf | 8. | 2472.33 | {1177., 4026.} | 87.5422 | 0.212807 | newly | 536. | 1095.92 | {504., 1780.} | 107.222 | 0.246545 | Reff | 0.0170047 | 0.198594 | {0.187163, 0.211698} | 1067.88 | ar | 99 380. | 93 958.7 | {93 807., 94 068.} | 5.45515 |
| 46 | R0 | {2.37755, 12.6461} | {2.9194, 10.1535} | {{2.9194, 10.1535}, {2.9194, 10.1535}} | {22.7903, 19.7104} | inf | 9. | 273.758 | {155., 412.} | 69.3096 | 0.548902 | newly | 377. | 146.323 | {83., 223.} | 61.6155 | 0.448846 | Reff | 0.0224678 | 0.192366 | {0.187163, 0.196738} | 756.183 | ar | 99 096. | 93 474.7 | {93 317., 93 647.} | 5.67255 |
| 47 | R0 | {1.69279, 11.6636} | {2.95376, 15.278} | {{2.95376, 15.278}, {2.95376, 15.278}} | {74.4906, 30.9887} | inf | 61. | 2230.66 | {1209., 3913.} | 92.806 | 0.233252 | newly | 550. | 856.814 | {455., 1491.} | 66.0031 | 0.182113 | Reff | 0.0676608 | 0.202051 | {0.192319, 0.215595} | 198.624 | ar | 96 214. | 93 714.1 | {93 543., 93 892.} | 2.5983 |
| 48 | R0 | {1.82402, 10.0038} | {2.9884, 12.8326} | {{2.9884, 12.8326}, {2.9884, 12.8326}} | {63.8359, 28.2773} | inf | 27. | 2136.16 | {790., 3324.} | 333.298 | 0.488804 | newly | 248. | 951.516 | {331., 1480.} | 283.858 | 0.433657 | Reff | 0.054447 | 0.196568 | {0.182233, 0.204317} | 261.026 | ar | 97 076. | 93 965.3 | {93 788., 94 129.} | 3.20439 |
| 49 | R0 | {1.6424, 11.1667} | {2.99939, 13.7076} | {{2.99939, 13.7076}, {2.99939, 13.7076}} | {82.6224, 22.7543} | inf | 29. | 936.522 | {437., 1575.} | 60.2484 | 0.182871 | newly | 321. | 390.029 | {178., 671.} | 51.8985 | 0.179723 | Reff | 0.0752383 | 0.184209 | {0.178704, 0.188932} | 144.834 | ar | 95 546. | 94 068.8 | {93 932., 94 197.} | 1.54606 |
| 50 | R0 | {2.05638, 14.3048} | {2.99939, 13.7076} | {{2.99939, 13.7076}, {2.99939, 13.7076}} | {45.8578, 4.17482} | inf | 38. | 1796.03 | {879., 3188.} | 41.7021 | 0.16173 | newly | 636. | 746.912 | {363., 1296.} | 42.9939 | 0.160507 | Reff | 0.0397498 | 0.189988 | {0.181913, 0.200299} | 377.958 | ar | 98 194. | 94 077.8 | {93 930., 94 214.} | 4.19193 |
